# Cost-effectiveness analysis of intravenous paricalcitol vs. oral calcitriol in the treatment of hyperparathyroidism secondary to chronic kidney disease

**DOI:** 10.1590/2175-8239-JBN-2022-0049en

**Published:** 2022-08-15

**Authors:** Marilia Mastrocolla de Almeida Cardoso, Juliana Machado-Rugolo, Silvana Andrea Molina Lima, Luis Gustavo Modelli de Andrade, Daniel da Silva Pereira Curado, Daniela Ponce

**Affiliations:** 1 Hospital das Clínicas da Faculdade de Medicina de Botucatu, Departamento de Gestão de Atividades Acadêmicas, Núcleo de Avaliação de Tecnologia em Saúde, Botucatu, SP, Brazil; 2 Ministério da Saúde, Departamento de Gestão e Incorporação de Tecnologias e Inovação em Saúde, Brasília, DF, Brazil

**Keywords:** Hyperparathyroidism, Secondary, Renal Insufficiency, Chronic, Paricalcitol, Calcitriol, Cost-Effectiveness Evaluation., Hiperparatireoidismo Secundário, Insuficiência Renal Crônica, Paricalcitol, Calcitriol, Avaliação de Custo-Efetividade

## Abstract

**Introduction:**

Hyperparathyroidism (SHPT) secondary to chronic kidney disease (CKD) is characterized by high levels of parathyroid hormone (PTH), hyperplasia of the parathyroid glands and cardiovascular disease. Selective and non-selective and selective vitamin D-receptor activators, calcimimetics, are available in the Brazilian market to reduce PTH levels.

**Objectives:**

To develop a cost-effectiveness (C/E) and budgetary impact (BI) analysis of intravenous paricalcitol vs. oral calcitriol for patients on dialysis with SHPT, from the perspective of the Brazilian Public Health Care System (SUS).

**Methodology:**

We built a decision-tree model to analyze C/E, which considered the outcome of avoided death and a time horizon of 1 year. As for the BI analysis, two scenarios were considered, one of demand and one of epidemiological approach, based on data from the Brazilian Society of Nephrology.

**Results:**

The analysis showed that the C/E ratio was R$ 1,213.68 per year, and an incremental effectiveness of 0.032, referring to avoided death. The incremental C/E ratio was R$37,927.50 per death averted by paricalcitol. It was estimated that the incremental BI with the expansion of paricalcitol use will be between R$1,600,202.28 and R$4,128,565.65 in the first year, considering the main and epidemiological scenarios. At the end of 5 years after the expansion of its use, an incremental BI was estimated between R$ 48,596,855.50 and R$ 62,90,555.73.

**Conclusion:**

Intravenous paricalcitol has superior efficacy and similar safety to oral calcitriol, reducing the overall mortality of dialysis patients, although it implies a higher cost.

## Resumo

**Introdução:**

O hiperparatireoidismo secundário (HPTS) à doença crônica renal (DRC) é caracterizado por elevados níveis de paratormônio (PTH), hiperplasia das glândulas paratireoides e doença cardiovascular. Para a redução dos níveis do PTH, estão disponíveis no mercado brasileiro os ativadores não seletivos e seletivos do receptor da vitamina D e os calcimiméticos.

**Objetivos:**

Desenvolver análise de custo-efetividade (C/E) e de impacto orçamentário (IO) do paricalcitol intravenoso vs. calcitriol oral para pacientes em diálise com HPTS, na perspectiva do Sistema Único de Saúde.

**Metodologia:**

Foi construído um modelo de árvore de decisão para a análise de C/E, que considerou o desfecho morte evitada e um horizonte temporal de 1 ano. Quanto à análise de IO, foram considerados dois cenários, sendo um de demanda aferida e um de abordagem epidemiológica, baseado nos dados da Sociedade Brasileira de Nefrologia.

**Resultados:**

A análise mostrou que a relação de C/E foi de R$ 1.213,68 ao ano, e uma efetividade incremental de 0,032, referente à morte evitada. A razão de C/E incremental foi de R$ 37.927,50 por morte evitada para o paricalcitol. Estimou-se que o IO incremental com a ampliação do uso do paricalcitol estará entre R$ 1.600.202,28 e R$ 4.128.565,65 no primeiro ano, considerando os cenários principal e o epidemiológico. Já no fim de 5 anos após a ampliação do uso, estimou-se IO incremental entre R$ 48.596.855,50 e R$ 62.90.555,73.

**Conclusão:**

O paricalcitol intravenoso tem eficácia superior e segurança semelhante ao comparador calcitriol oral, diminuindo a mortalidade geral dos pacientes em diálise, embora implique maior custo.

## Introduction

Hyperparathyroidism (SHPT) secondary to chronic kidney disease (CKD) is characterized by high serum levels of parathyroid hormone (PTH), parathyroid gland hyperplasia, high remodeling bone disease and cardiovascular disease^1^. The serum PTH level considered adequate for patients with stage 5D CKD is between 150 and 300 pg/mL or two to nine times the limit value of the dosage method^2,3^. According to the census of the Brazilian Society of Nephrology (SBN), in 2020, it was estimated that 144,779 patients were on dialysis in Brazil^4^. Of these, approximately 18% had PTH levels above 600 pg/mL in 2019, while in 2014 they were around 26%, suggesting that there was some impact on reducing PTH levels by taking paricalcitol and cinacalcet and implementation of the PCDT in 2017^5^. To reduce PTH levels, there are three classes of drugs available in the Brazilian market: non-selective vitamin-D receptor activators (calcitriol and alfacalcidol), selective VDR activators (paricalcitol) and calcimimetics (cinacalcet hydrochloride)^5^. Among the aforementioned drugs, SUS provides oral calcitriol, and its intravenous presentation was discontinued in 2020, and oral alfacalcidol, in 2017. The availability of paricalcitol in the SUS is aimed at patients with PTH equal to or greater than 500 pg/mL and, for cinacalcet, to patients with PTH levels above 800 pg/mL^6^. In this sense, the objective of this document was to develop a cost-effectiveness and budgetary impact analysis of paricalcitol vs. oral calcitriol for dialysis patients with SHPT, from the perspective of the Brazilian Public Healthcare System (SUS) after analyzing new existing scientific evidence on the use of paricalcitol, aiming at its expansion of use for the treatment of SHPT associated with stage 5D CKD in the following scenarios: 1) first line for patients with PTH > 300 pg/mL in the absence of hyperphosphatemia and hypercalcemia; 2) as a replacement for cinacalcet in patients who have the adverse effects of hypocalcemia without improvement after adjusting the calcium concentration of the dialysate and reducing the dose of cinacalcet; 3) in association with cinacalcet in patients who did not reach target PTH levels (< 300 pg/mL).

## Methodology

We searched for evidence in The Cochrane Library, MedLine (via PubMed), Embase (Elsevier), PubMed Central, Epistemonikos, NICE and Virtual Health Library databases. Finally, we included the systematic review by Geng et al.^7^, published in 2020 in Plos One, for the synthesis of evidence. This review aimed to assess the safety and efficacy of paricalcitol vs. non-selective vitamin-D analogues in the management of SHPT in patients with CKD 5D regarding PTH, calcium, phosphorus levels and adverse events. Fifteen studies were included for meta-analysis: 11 randomized controlled trials (RCTs), eight of which compared paricalcitol vs. calcitriol, 1 RCT comparing paricalcitol vs. maxacalcidol, 1 RCT comparing paricalcitol vs. alfacalcidol, and 1 RCT comparing paricalcitol vs. cinacalcet; 3 quasi-experimental studies (NRSI) comparing paricalcitol vs. calcitriol; and 1 NRSI comparing paricalcitol vs. calcitriol/doxercalciferol, totaling 110,544 patients with stage 5D CKD. Methodological quality was rated as moderate, based on the 16 domains of the AMSTAR-28.

Regarding clinical outcomes, all-cause mortality of patients treated with paricalcitol was lower than that of patients receiving non-selective vitamin-D analogues, with an RR of 0.84 (95% CI 0.79-0.90 ; p <0.001), according to the meta-analysis performed by the group, which included only studies that compared paricalcitol vs. calcitriol found in RS by Geng et al.^7^. There were no significant differences in the incidence of adverse events such as hypercalcemia, hyperphosphatemia and PTH levels. In addition, most outcomes had low quality of evidence, with the exception of mortality, which was moderate.

## Economic assessment

Based on literature data, we ran an economic assessment to estimate the incremental cost-effectiveness ratio (ICER) of paricalcitol compared to oral calcitriol for the treatment of hyperparathyroidism secondary to stage 5D chronic kidney disease. The study design followed the premises of the Methodological Guidelines for Economic Evaluation of the Ministry of Health^9^. In order to increase the transparency of the proposed study, the main aspects of the studies were summarized according to the CHEERS Task Force Report^10^ checklist.

### Estimate of resources and costs

In consultation with the Healthcare Price Database (BPS), a purchase made by the Health Logistics Department of the Ministry of Health (DLOG/MS) in the period between 04/04/2020 to 10/04/2021 was fund in the amount of R $16.50 per unit of the drug paricalcitol. For calcitriol, we used the value of R$1.09, referring to the weighted average of the price practiced in public purchases carried out in the last 18 months, according to the Integrated System of General Services Administration (SIASG), since no purchases were made by the DLOG/MS.

For oral calcitriol, a dose of 2 mcg on alternate days was considered (6 mcg/week divided into 3 dialysis sessions), and for injectable paricalcitol it would be 5 mcg on alternate days (15 mcg/week divided into 3 dialysis sessions), using a 1:2.5 ratio of calcitriol to paricalcitol. To estimate drug costs, the value of R$16.50 was used for the unit of paricalcitol (5 ucg/mL vial), considering the identification of a purchase made by DLOG/MS, and for Calcitriol was used as the weighted average (R$ 1.09 per 0.25 ucg capsule) of the price practiced in public purchases carried out in the last 18 months, both checked at the BPS. Other direct costs, such as consultations and laboratory tests, were not considered.

Chart 1 shows the average monthly and annual cost of intravenous paricalcitol and oral calcitriol drugs per patient.

**Chart 1:** Mean weekly cost of intravenous paricalcitol and oral calcitriol per patient

Medication	Unit price	Dose	Weekly use	Weekly cost (per patient)	Annual cost (per patient)
Paricalcitol 5ucg/mL (vial)	R$ 16.50	0.04 - 0.1 ucg/kg/dose	3 vials	R$ 49.50	R$ 2,574.00
Oral Calcitriol 0.25 ucg	R$1.09	2 ucg/alternate days	24 pills	R$26.16	R$ 1,360.32

### Efficiency

The probabilities of transition between states (dialysis and death) were obtained from the literature and from the 2020 Brazilian Dialysis Census published by SBN, and mortality was estimated at 20% per year3,4. For the paricalcitol group, the RR of mortality was 0.84 (95% CI, 0.79-0.90), that is, a reduction of 16%, according to the SR and meta-analyses considered in the preparation of this report^5,6^, considering the 16.8% mortality rate in the paricalcitol group.

### Economic model

**Figure 1:**
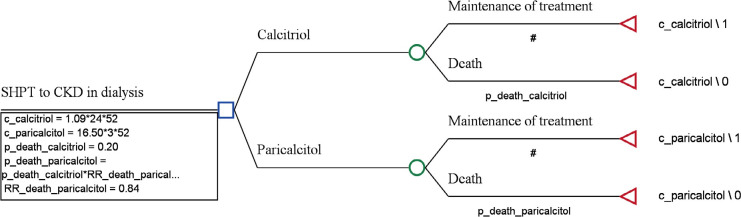
Decision-tree for cost-effectiveness analysis. Legend: C_calcitriol: 1.09 (cost per unit of calcitriol)* 24 (weekly dose)* 52 (number of weeks in the year); C_paricalcitol: 16.50 (cost per unit of paricalcitol)* 3 (weekly dose)* 52 (number of weeks in the year); Annual death probability_calcitriol = 0.20; RR_death_Paricalcitol = 0.84 (16% reduction).

The analytical model adopted was the decision tree to conduct the economic evaluation in the TreeAge Pro 2009 software. The simple decision tree was used to build the economic model. In the decision, two possibilities were considered: dialysis while using the drug and death. The decision tree format is shown in Figure 1.

## Budget impact analysis

We ran an analysis to estimate the budgetary impact of expanding the use of paricalcitol, in the SUS, for the treatment of SHPT to CKD in dialysis patients.

The analysis of the budget impact adopted the perspective of the Brazilian Public Healthcare System (SUS), as it is the holder of the budget at the federal level, as recommended by the Methodological Guideline for Budget Impact Analysis of the Ministry of Health (MS)^9^.

The time horizon used was 5 years, according to MS Guidelines.

## Population

Three scenarios were considered: the main one of measured demand, based on data from the Department of Pharmaceutical Assistance and Strategic Inputs of the Ministry of Health (DAF); the alternative of measured demand, based on data from the Open Room of Health Intelligence (SABEIS)^11^; and the epidemiological alternative, based on data from the Brazilian Society of Nephrology (SBN).

According to the main scenario, DAF data show that, in 2020, 14,138 patients used paricalcitol (9.8% of the population on dialysis). With the expansion of use, it is estimated that half of the patients using oral calcitriol would be indicated for paricalcitol because they persist with PTH levels above 300 pg/mL, in the absence of hypercalcemia and hyperphosphatemia, increasing to 20% of patients on dialysis the use of paricalcitol.

According to the alternative scenario of measured demand, data from SABEIS show that, in 2020, around 13.5% of patients used paricalcitol (19,326 patients). With the expansion of use, it is estimated that half of the patients who use oral calcitriol would be indicated for paricalcitol because they persist with PTH levels above 300 pg/mL, in the absence of hypercalcemia and hyperphosphatemia, increasing to 23% of patients on dialysis the use of paricalcitol.

For the alternative scenario of the epidemiological approach, the prevalent dialysis population of 144,779 patients was considered, according to the SBN Dialysis Census, 2020, with an annual growth of the dialysis population of 5%. Of these, around 18% of patients have moderate SHPT (PTH above 600 pg/mL) (SBN, 2019), which totals 26,060 patients with a potential indication of paricalcitol use, provided that calcium and phosphorus levels were controlled. According to SBN epidemiological data, around 4.9% of patients were using paricalcitol; 4.4% were using intravenous calcitriol; and 20% were using oral calcitriol in 2020. Given that intravenous calcitriol was discontinued in 2021, even without the expansion of the use of paricalcitol, these patients were considered to have migrated to paricalcitol. Thus, the population in use would increase from 4.9% to 9.3%.

With the expansion of use, it is estimated that half of the patients who use oral calcitriol would be indicated for paricalcitol because they persist with PTH levels above 300 pg/mL, in the absence of hypercalcemia and hyperphosphatemia, increasing to 20% the use of paricalcitol by patients on dialysis.

## Results

### Cost-effectiveness assessment

The analysis showed that the use of paricalcitol results in an incremental cost of R$ 1,213.68 per year and an incremental effectiveness of 0.032, referring to the deaths avoided in one year. The ICER was R$ 37,927.50 per death averted for paricalcitol.

### Budget impact analysis

#### Main scenario - DAF data (measured demand)

In the main scenario, considering DAF data for a measured demand, an incremental budget impact was estimated with the expansion of paricalcitol use of BRL 4,128,565.65 in the first year, and BRL 62,290,555.73 at the end of five years (Table 1).

**Table 1:** Budget impact in 5 years concerning the treatment of HPTS in CKD in the dialysis population using vitamin D analogues with the expansion of paricalcitol use (DAF setting)

Year	Eligible population	Budgetary impact with oral calcitriol^*^ (Base scenario)	Diffusion rate for paricalcitol	Budgetary impact with paricalcitol^**^ and calcitriol^*^ (proposed scenario)	Incremental budgetary impact with paricalcitol
2021	27.363	R$ 73.574.279,71	12%	R$ 77.702.845,36	R$ 4.128.565,65
2022	25.539	R$77.253.526,08	14%	R$ 85.165.059,46	R$ 7.911.533,38
2023	23.464	R$ 81.116.202,38	16%	R$ 93.178.647,48	R$ 12.062.445,10
2024	21.118	R$ 85.171.988,30	18%	R$ 101.780.652,73	R$ 16.608.663,43
2025	18.478	R$ 89.430.563,52	20%	R$ 111.009.911,04	R$ 21.579.347,52
**Total in 5 years**		**R$ 406.546.560,00**	-	**R$ 468.837.115,73**	**R$ 62.290.555,73**

* Annual cost of treatment with oral calcitriol per patient = R$ 1260,32;

** Annual cost of treatment with paricalcitol, per patient = R$ 2,574.00.

DAF: pharmacy assistance department; CKD: chronic kidney disease; SHPT: second hyperparathyroidism.

#### Alternative scenario - knowledgeable data (measured demand)

In the alternative scenario that considered the measured demand data from SABEIS, there was an incremental budget impact with the expansion of paricalcitol use of R$ 1,600,202.28 in the first year, and R$ 48,596,855.50 at the end of five years (Table 2).

**Table 2:** 5-year budgetary impact concerning the treatment of SHPT on CKD for the dialysis population using vitamin-D analogues with paricalcitol use expansion (SABEIS scenarios)

Year	Eligible population	Budgetary impact with oral calcitriol^*^ (Base scenario)	Diffusion rate for paricalcitol	Budgetary impact with paricalcitol^**^ and calcitriol^*^ (proposed scenario)	Incremental budgetary impact with paricalcitol
2021	27.363	R$ 86.938.486,13	15%	R$ 88.538.688,40	R$ 1.600.202,28
2022	27.135	R$ 91.286.039,52	16%	R$ 94.754.518,27	R$ 3.468.478,85
2023	25.140	R$ 95.850.341,50	18%	R$ 103.247.579,23	R$ 7.397.237,73
2024	17.598	R$ 100.642.829,98	23%	R$ 118.267.679,09	R$ 17.624.850,11
2025	18.478	R$ 105.674.942,88	23%	R$ 124.181.029,44	R$18.506.086,56
**Total in 5 years**		**R$ 480.392.640,01**	-	**R$ 528.989.495,51**	**R$ 48.596.855,50**

* Annual cost of treatment with oral calcitriol per patient = R$ 1.360,32;

** Annual cost of treatment with paricalcitol, per patient = R$ 2.574,00. DAF: pharmacy assistance department; CKD: chronic kidney disease; SHPT: second hyperparathyroidism.

#### Alternative scenario - SBN data (epidemiological)

Table 3 shows the budgetary impact of the epidemiological scenario without increasing use and with increasing use of paricalcitol in 1 to 5 years. It was estimated that the incremental budget impact of increasing the use of paricalcitol will be BRL 4,128,565.65 in the first year, and BRL 62,290,555.73 at the end of five years (Table 3).

**Table 3:** Budget impact in 5 years concerning the treatment of HPTS in CKD in the dialysis population using vitamin D analogues with the expansion of paricalcitol use (epidemiological setting)

Year	Eligible population	Budgetary impact with oral calcitriol^*^ (Base scenario)	Diffusion rate for paricalcitol	Budgetary impact with paricalcitol^**^ and calcitriol^*^ (proposed scenario)	Incremental budgetary impact with paricalcitol
2021	27.363	R$ 73.574.279,71	12%	R$ 77.702.845,36	R$ 4.128.565,65
2022	25.539	R$77.253.526,08	14%	R$ 85.165.059,46	R$ 7.911.533,38
2023	23.464	R$ 81.116.202,38	16%	R$ 93.178.647,48	R$ 12.062.445,10
2024	21.117	R$ 85.171.988,30	18%	R$ 101.780.652,73	R$ 16.608.663,43
2025	18.478	R$ 89.430.563,52	20%	R$ 111.009.911,04	R$ 21.579.347,52
**Total in 5 years**		**R$ 406.546.560,00**	-	**R$ 468.837.115,73**	**R$ 62.290.555,73**

* Annual cost of treatment with oral calcitriol per patient = R$ 1.360,32;

** Annual cost of treatment with paricalcitol, per patient = R$ 2.574,00. CKD: chronic kidney disease; SHPT: second hyperparathyroidism.

We estimated that the incremental budget impact with the expansion paricalcitol use in the SUS will be between R$ 1,600,202.28 and R$ 4,128,565.65 in the first year, considering the main scenarios, based on DAF and SABEIS data^10^, and the epidemiological one, based on data from the SBN. After 5 years of use expansion, an incremental impact was estimated to range from R$48,596,855.50 to R$62,90,555.73, depending on the scenario considered.

## Discussion

In this study, SHPT patients with stage 5 CKD were evaluated with the aim of performing a cost-effectiveness analysis and budgetary impact of paricalcitol versus oral calcitriol, from the perspective of the Brazilian Public Healthcare System. We chose not to develop a Markov decision model because the time horizon chosen was one year.

We included the systematic review by Geng et al.^7^, published in 2020 in Plos One, for the synthesis of evidence, since it is aimed to assess the safety and efficacy of paricalcitol vs. calcitriol in the management of SHPT in patients with CKD 5D regarding PTH, calcium, phosphorus levels and adverse events. Regarding clinical outcomes, all-cause mortality in patients treated with paricalcitol was lower when compared to mortality in patients who received calcitriol (RR=0.84; 95% CI 0.79-0.90; p<0.001). There were no significant differences in the incidence of adverse events such as hypercalcemia and hyperphosphatemia and in PTH levels.

Although without additional benefits in terms of PTH and phosphorus levels and the need for parathyroidectomy, we concluded that paricalcitol has superior efficacy and similar safety to oral calcitriol, decreasing the risk of mortality in dialysis patients. In view of this evidence, for the cost-effectiveness analysis, the outcome of death avoided was considered. As a result of the comparison of oral paricalcitol versus oral calcitriol from the perspective of the SUS, the cost-effectiveness analysis showed that the use of paricalcitol results in an incremental cost of R$ 1,213.68 per year, and an incremental effectiveness of 0.032 in terms of avoided death in a year. The ICER was BRL 37,927.50 per death averted by paricalcitol. This ICER value is acceptable for Brazil, whose cost-effectiveness threshold is around BRL 81,675.00 per increase in QALY12.

As for the IBI, it was estimated that the incremental budget impact with the expansion of the use of paricalcitol in the SUS will be between R$ 1,600,202.28 and R$ 4,128,565.65 in the first year, considering the main scenarios, based on DAF and SABEIS data, and epidemiological data - SBN data. After 5 years of use expansion, an incremental impact was estimated to range from R$48,596,855.50 to R$62,90,555.73, depending on the scenario considered.

The main limitation of the study carried out concerns the estimation of the target population, which was estimated based on data from SBN records. Although there are epidemiological data on the population on dialysis, with SHPT to CKD and with levels of PTH, calcium and phosphorus above the target, they are estimated data, based on records, which may be overestimated, considering that we have approximately 40% of Brazilian centers of dialysis participants in the 2020 Census, most of them universities. This hypothesis is strengthened when we compare the epidemiological data from the SBN with the real data from the DAF, which are 20% lower than the data reported by the SBN, but close to the SABEIS data.

Given that intravenous calcitriol was discontinued in 2021, even without scaling up paricalcitol use, patients were considered to have migrated to paricalcitol, leading to increased drug cost estimat es, as the population in use would increase from 4.9% to 9.3%.

Another limitation of the study is the extrapolation of mortality reduction from data from a systematic literature review. Furthermore, the systematic review performed by Geng et al.^7^ includes very heterogeneous studies.

Another point to highlight is that the rate of diffusion predicted in the three scenarios was defined through assumptions related to the probable future use of paricalcitol in the SUS, which is still very uncertain. Finally, another limitation of the AIO is the lack of knowledge of the number of patients with contraindication to the use of paricalcitol and the failure to obtain the number of patients using calcitriol by DAF or SABEIS, since the drug is also dispensed for other ICDs.

## Conclusion

As a result of the comparison between paricalcitol and oral calcitriol from the perspective of the SUS, the analysis showed that the cost-effectiveness ratio (C/E) was R$ 1,213.68 per year with an incremental effectiveness of 0.032, referring to the death avoided in a year. The incremental C/E ratio was R$37,927.50 per death averted for paricalcitol. It was estimated that the incremental budget impact of expanding the use of cinacalcet in the SUS will be between R$1,600,202.28 and R$4,128,565.65 in the first, considering the main and epidemiological scenarios based on SBN data. At the end of 5 years after the expansion of use, an incremental impact between R$ 48,596,855.50 and R$ 62,90,555.73 was estimated; considering the same scenarios. Therefore, paricalcitol has superior efficacy and similar safety to calcitriol, reducing the overall mortality of patients on dialysis, and it can be considered cost-effective for the SUS.
